# [^18^F]FES uptake in the pituitary gland and white matter of the brain

**DOI:** 10.1007/s00259-021-05281-8

**Published:** 2021-03-17

**Authors:** R. Iqbal, C. W. Menke-van der Houven van Oordt, D. E. Oprea-Lager, J. Booij

**Affiliations:** 1 Department of Medical Oncology, Cancer Center Amsterdam, Amsterdam UMC, Vrije Universiteit Amsterdam, De Boelelaan, 1117 Amsterdam, Netherlands; 2 Department of Radiology and Nuclear Medicine, Cancer Center Amsterdam, Amsterdam UMC, Vrije Universiteit Amsterdam, De Boelelaan, 1117 Amsterdam, Netherlands; 3 Department of Radiology and Nuclear Medicine, Cancer Center Amsterdam, Amsterdam UMC, Academisch Medisch Centrum, Meibergdreef 9, Amsterdam, Netherlands

16α-[^18^F]-fluoro-17β-estradiol ([^18^F]FES) is a positron emission tomography (PET) tracer, developed for in vivo visualization of the estrogen receptor (ER). It is widely used in oncology, mostly in patients with breast cancer, to identify ER positive lesions. However, besides its application in oncology, [^18^F]FES has also been evaluated for assessment of ER expression in the brain [1]. This has been investigated in rats where high specific [^18^F]FES uptake could be observed in regions with high ER density in the brain, i.e., the pituitary gland and hypothalamus [1]. Hattersley et al. confirmed this clinically in healthy post-menopausal women [2]. Strikingly, this study showed that [^18^F]FES also accumulates in white matter [2], as was also found in a case of our recent study (NCT03726931) and depicted in the Figure. A 71-year-old post-menopausal woman with ER-positive breast cancer underwent [^18^F]FES PET/CT imaging (Figure: pituitary gland and white matter on PET (**a/d**), low-dose CT (**b**/**e**), and fused PET/CT (**c**/**f**) images). The Figure shows the characteristic uptake of the radiotracer in the pituitary gland (SUV_max_: 1.88, SUV_mean_: 1.44) and in white matter (SUV_max_:1.36, SUV_mean_: 1.16). Interestingly, in the study performed by Hattersley et al., [^18^F]FES uptake in the pituitary gland could be blocked with an ER-antagonist, while uptake in the white matter could not, suggesting that [^18^F]FES uptake in white matter reflects non-specific uptake [1, 2]. The present finding requires awareness in clinical practice as it suggests that [^18^F]FES uptake in the pituitary gland and white matter occurs physiologically and that it does not necessarily indicate pathological uptake of the tracer.

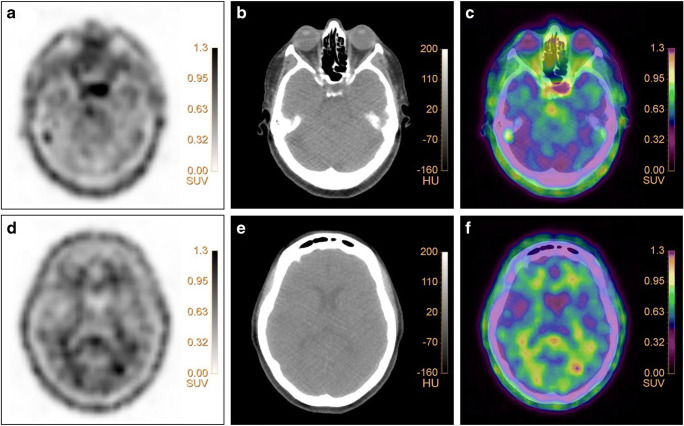



## Data Availability

The data supporting the conclusions of this article is included within the article.
